# Stimulatory Action of Cyclooxygenase Inhibitors on Hematopoiesis: A Review

**DOI:** 10.3390/molecules17055615

**Published:** 2012-05-10

**Authors:** Michal Hofer, Milan Pospíšil, Zuzana Hoferová, Lenka Weiterová, Denisa Komůrková

**Affiliations:** Laboratory of Experimental Hematology, Institute of Biophysics, v.v.i., Academy of Sciences of the Czech Republic, Královopolská 135, CZ-61265 Brno, Czech Republic; Email: ksoucek@ibp.cz (M.P.); hoferovaz@centrum.cz (Z.H.); weiterova@seznam.cz (L.W.); komurkovad@seznam.cz (D.K.)

**Keywords:** prostaglandins, cyclooxygenase inhibition, cycloxygenase-2 inhibitors, hematopoiesis, myelosuppression

## Abstract

The presented review summarizes experimental data obtained with a mouse model when investigating the relationship between inhibition of prostaglandin production and hematopoiesis. While prostaglandin E_2_ acts in a negative feedback control of myelopoiesis, inhibition of cyclooxygenases, responsible for its production, shifts the feedback to positive control. Based on these relationships, agents inhibiting cyclo-oxygenases, known as non-steroidal anti-inflammatory drugs (NSAIDs), can activate hematopoiesis and be protective or curative under myelosuppressive states. The effectiveness of therapeutic use of NSAIDs in these situations is expressive especially under the selective inhibition of cyclooxygenase-2 (COX-2), when undesirable side effects of cyclooxygenase-1 inhibition, like gastrointestinal damage, are absent. The effects of the clinically approved selective COX-2 inhibitor, meloxicam, were investigated and demonstrated significant hematopoiesis-stimulating and survival-enhancing actions of this drug in sublethally or lethally γ-irradiated mice. These effects were connected with the ability of meloxicam to increase serum levels of the granulocyte colony-stimulating factor. It can be inferred from these findings that selective COX-2 inhibitors might find their use in the treatment of myelosuppressions of various etiologies.

## 1. Introduction

Hematopoiesis is an intricate complex of processes through which functional blood cells of several lineages are produced. These processes take place in hematopoietic tissues which are a compartmentalized system of cells undergoing proliferation and differentiation during their development from stem cells through progenitor and precursor cells resulting in production of mature blood cells. These proliferation and differentiation processes are regulated by a number of growth factors, cytokines and other molecules [1,2].

Among these molecules and processes, an important role is played by prostaglandins and by inhibition of their production via the action of cyclooxygenases. Cyclooxygenases, also known as prostaglandin H-2 synthases, are heme-containing enzymes which catalyze the first committed step in prostaglandin synthesis. They catalyze two distinct reactions, namely cyclization of arachidonic acid to form prostaglandin G_2_ and hydroperoxidation of prostaglandin G_2_ to form prostaglandin H_2_ [3,4,5]. Prostaglandin H_2_ is subsequently converted to biologically active products including, among others, prostaglandin E_2_ (PGE_2_), which participates in the regulation of hematopoiesis and plays an important role in the negative hematopoietic feedback control [6,7,8,9,10,11]. On the other hand, inhibition of PGE_2_ production shifts the regulatory balance to positive control. Due to these mechanisms the pharmacological inhibition of cyclooxygenases by agents known as non-steroidal anti-inflammatory drugs (NSAIDs) offers the possibility to positively influence hematopoietic functions. NSAIDs are employed clinically not only for treatment of inflammatory states [12], but also in other indications like the prevention [13] and treatment of cancer [14]. The possibilities of cancer treatment may be closely related to hematological effects of cyclooxygenase inhibitors, summarized in this review, because the suppression of hematopoiesis belongs to frequent undesirable side effects of oncological therapy utilizing cytotoxic drugs. These aspects substantiate investigation of hematopoiesis-modulating action of cyclooxygenase inhibitors in experimental studies.

It should be mentioned that PGE_2_ was reported to promote hematopoietic stem cell proliferation [15], as well as stem cell renewal and engraftment [16,17]. These findings seem to be in contradiction to numerous findings on suppressor effects of PGE_2_ on hematopoiesis and on hematopoiesis-stimulating action of cyclooxygenase inhibitors. Their explanation in connection with the reported stimulatory effects of NSAIDs may consist in the fact that the hematopoietic stem cells compartment does not play a determinant role in the time period of the lowest hematopoietic depression following a myelo-suppressive intervention. In these situations the condition of the more differentiated compartments of hematopoietic progenitor and precursor cells plays a key role in regeneration of hematopoiesis. Moreover, the described PGE_2_-induced enhanced stem cell renewal [16,17] may reflect a decreased outflow of cells to the more differentiated compartments where their presence is indispensable for successful hematopoietic recovery. Timing and dosing effects of the drug administration have to be taken into account, as well.

Since cyclooxygenases carrying out prostaglandin synthesis exist in two isoforms, namely cyclooxygenase-1 (COX-1), which is expressed constitutively in a variety of tissues including the gastrointestinal tract, and cyclooxygenase-2 (COX-2), which is inducible and responsible for the production of prostaglandins during inflammatory states [18,19,20], two types of cyclooxygenase-inhibiting drugs can be distinguished. The first type comprises non-selective cyclooxygenase inhibitors which affect both COX-1 and COX-2 [18], the second type is represented by selective COX-2 inhibitors which are devoid of the undesirable effects induced by the inhibition of COX-1 and are responsible, e.g., for gastrointestinal damage [21]. Experimental and clinical data demonstrate a reduced risk of the undesirable gastrointestinal side effects after administration of COX-2-selective inhibitors as compared with the effects induced by classical non-selective cyclooxygenase inhibitors [22].

## 2. Action of Non-Selective Cyclooxygenase Inhibitors on Hematopoiesis: Desirable and Undesirable Effects of the Treatments— The History

During the eighties of the last century, prostaglandins were shown to inhibit the proliferation of hematopoietic progenitor cells [6]. In addition, the administration of indomethacin, a drug belonging to the NSAID class and acting on the principle of non-selective inhibition of cyclooxygenases, was observed to induce mouse splenic erythropoiesis and to increase numbers of granulocyte-macrophage progenitor cells in bone marrow [8]. These and other findings in mice enabled formulation of the hypothesis that prostaglandins, especially those of the E type, played a negative feedback factor role in regulation of granulopoiesis and proliferation of more primitive hematopoietic cells [9,10,11]. As concerns detailed mechanisms of the effects observed, it was found that monocytes/macrophages were producers of prostaglandins as well as of regulatory cytokines and hematopoietic growth factors [23] and that the production of cytokines by macrophages could be modulated by exogenous prostaglandins [24] and by administration of NSAIDs [25,26]. These observations strongly suggested that an indirect mechanism, *i.e*., mediation through cells not directly involved in hematopoietic lineages directed at production of erythrocytes, granulocytes, lymphocytes or platelets, played a key role in the modulation of hematopoiesis via the regulatory feedback system mediated by prostaglandins.

Various non-selective cyclooxygenase inhibitors were reported to stimulate proliferation of hematopoietic stem cells [27], to enhance bone marrow and splenic hematopoiesis [28,29], and bone marrow erythropoiesis [30] in mice. Many studies have been performed by several research groups on the topic of experimental treatment of hematopoiesis suppressed by ionizing radiation (described in more detail in an earlier review [31]). Positive therapeutical outcomes, manifested as an enhancement of murine hematopoiesis after radiation damage, were reported when NSAIDs (indomethacin, diclofenac, or flurbiprofen) were administered before [32,33,34,35] or after irradiation [36,37,38], as well as when given to mice exposed to fractionated irradiation [39,40,41], to continuously irradiated rats [42], or lethally irradiated mice who received syngeneic bone marrow transplantation [43,44]. Administration of diclofenac was found to stimulate hematopoiesis also in tumor-bearing mice [45] and NSAIDs were observed to improve the processes of hematopoietic regeneration following exposures of experimental mice to ionizing radiation when administered concomitantly with immunomodulators glucan [46,47], muramyl tripeptide phosphatidylethanolamine [48,49,50], or broncho-vaxom [51,52], as well as with chemical radioprotectors cystamine or WR-2721 [53,54,55,56]. An improvement of hematopoietic recovery after administration of non-selective cyclooxygenase inhibitors was also observed in mice whose bone marrow was damaged by cytotoxic anti-tumor drugs [57,58,59]. The above-mentioned studies have shown that non-selective inhibitors of cyclooxygenases enhance a wide spectrum of hematological parameters in experimental animals, ranging from multipotent progenitor cells to mature blood cells.

However, as already briefly stated in the Introduction, it is known from experimental investigations [60] as well as from clinical observations [61] that administration of non-selective cycloxygenase inhibitors, called sometimes classical NSAIDs, is frequently accompanied by undesirable side effects, especially those in the gastrointestinal tract. These effects arise from the ability of prostaglandins to act cytoprotectively in the gastrointestinal mucosa [62]. In connection with the above-summarized findings of stimulatory effects of classical NSAIDs on radiation-suppressed hematopoiesis, it should be mentioned that these observations were carried out after sublethal irradiation inducing a pure bone marrow syndrome of the acute radiation disease without any serious damage to the gastrointestinal tract. However, after exposure to lethal radiation doses, damaging both the bone marrow and the gastrointestinal tissues, a reduced survival and appearance of early deaths were found in mice treated with classical NSAIDs either before [63] or after irradiation [64]. Histological examination revealed pathological changes in the NSAIDs-treated mice, including a partial loss of intestinal epithelium [64]. With the aim to obtain NSAIDs with reduced gastrointestinal side effects, a number of derivatives of classical NSAIDs were synthesized and tested. It was found out that a nitroderivative of commonly clinically used NSAID flurbiprofen, flurbiprofen 4-nitroxybutylester [65], exhibiting reduced ulcerogenic properties, retained the hematopoiesis-stimulating effectiveness of the original drug and enhanced insignificantly survival after lethal irradiation [66].

The results obtained in studies on hematological and/or radioprotective effects of non-selective cyclooxygenase inhibitors have been evaluated by Michalowski [67] who summarized that “Further studies on anti-inflammatory drug treatment of radiation damage to normal organs are justified and desirable”.

## 3. Action of Selective Cyclooxygenase-2 Inhibitors on Hematopoiesis: Desired Shift in the Treatment Outcome—Recent Findings

Because of the low incidence and intensity of their gastrointestinal side effects, selective COX-2 inhibitors also became interesting for investigation of their possible hematopoiesis-stimulating action. If they retained the stimulatory effectiveness of the classical NSAIDs, they could be used advantageously in the treatment of both main indications of myelosuppression, namely in patients with the bone marrow suppression induced by radiation exposure and in those with the hematopoiesis damaged by cytotoxic anti-cancer drugs.

First attempts to evaluate the hematopoiesis-modulating abilities of the selective COX-2 inhibition were performed in the 1990’s when a significant increase in counts of total white blood cells and neutrophils in experimentally burned rats treated with the selective COX-2 inhibitor NS-398 was reported [68].

During the last six years, a promising aggregate of findings has been accumulated indicating the hematopoiesis- and survival-modulating action of meloxicam, another clinically available selective COX-2 inhibitor [69]. In 2006, the authors of this review published their findings showing that meloxicam stimulates both granulopoiesis and erythropoiesis in mice receiving the drug either in a single dose before whole-body irradiation with a dose of 6.5 Gy γ-rays or repeatedly (four times) after irradiation with a dose of 4 Gy [70]. Another study has shown that meloxicam given to mice either singly or repeatedly after a sublethal irradiation increases the level of serum granulocyte colony-stimulating factor (G-CSF) [71]; thus, the induction of G-CSF production by meloxicam can be considered an important mechanism of its hematopoiesis-stimulating effectiveness. Positive effects of administration of meloxicam on radiation-suppressed hematopoiesis have been confirmed also by investigations of mice irradiated with mid-lethal doses of 7.50 or 7.75 Gy γ-rays; a significantly improved survival and significant elevation of important hematological parameters in mice administered the selective COX-2 inhibitor 1 h before irradiation has been recorded [72]. The ability of meloxicam to elevate serum G-CSF levels has been confirmed in this study as well. Jiao *et al*. [73] have brought attention to the loss of survival-enhancing effects of meloxicam if the drug was administered repeatedly (seven times) in the post-irradiation period following a lethal radiation exposure. However, our recent studies [74] have demonstrated that a mere single dose of meloxicam given to mice shortly (1 h) after a lethal irradiation is sufficient for obtaining the desirable survival-increasing effects. Also this simple pharmacological treatment has been accompanied by an elevation of serum G-CSF [74]. The loss of positive effects of meloxicam after its repeated post-irradiation administration [73] can be evoked by vascular [75] or hepatic [76] side effects of meloxicam which can manifest themselves easily under the conditions of a serious post-irradiation stress. On the other hand, the positive effect of the single early post-irradiation injection of meloxicam [74] is in agreement with the demand for a very early post-irradiation therapeutic intervention, as proposed by Hérodin and Drouet [77,78]. Meloxicam has been shown to act positively also in a combined treatment regimen when given in a single early post-irradiation dose followed by two post-irradiation injections of IB-MECA, an adenosine A_3_ receptor agonist [79]. Taken together, the results of the studies on the action of the selective inhibitor of cyclooxygenase-2, meloxicam, clearly show that this drug retains the hematopoiesis-stimulating effects of the classical NSAIDs but shows much less undesirable side effects and, thus, they strongly suggest the possibility of its use in the treatment of radiation-induced myelosuppression.

As concerns the mechanisms of meloxicam action, the described ability of meloxicam to elevate serum levels of G-CSF bears evidence for the assumption that meloxicam does not stimulate hematopoietic cells directly but that at least a part of its stimulatory effects on proliferation of hematopoietic progenitor and precursor cells is indirect, mediated through its interaction with G-CSF-producing cells. A seemingly contradictory finding on the role of PGE_2_ and production of G-CSF was reported concerning the ability of PGE_2_ to stimulate LPS-primed G-CSF release from mouse peritoneal neutrophils [80]. However, the authors of this report themselves interpret their finding as a result of regulation of local production of G-CSF during an acute inflammation, which represents an expressive difference in comparison with our observations of a system G-CSF elevation and action achieved by administration of a COX-2 inhibitor.

## 4. Notes on the Use of Meloxicam in Anti-Tumor Therapy in Connection with Its Hematopoiesis-Stimulating Action

Meloxicam has been repeatedly reported to show anti-tumor activities [81,82,83,84,85,86]. With regard to the above-summarized findings of stimulatory effects of meloxicam on radiation-suppressed hematopoiesis, it is justified to assume that meloxicam should similarly support also hematopoiesis damaged by cytotoxic drugs. Nevertheless, further investigations testing this assumption are needed. Induction of combined anti-tumor and hematopoiesis-stimulating effects in one drug would be very advantageous.

Moreover, the effects of combined administration of meloxicam or other selective COX-2 inhibitors with hematopoietic growth factors (e.g., G-CSF) should be studied in detail, as well. G-CSF is a rather costly drug [87]. The above-mentioned findings bearing evidence of the ability of meloxicam to induce the production of G-CSF [71,72,74] suggest that a part of the total dose of G-CSF could be substituted by a cheaper drug like meloxicam.

## 5. Conclusions

Based on the results presented in this review, its authors are of the opinion that clinical studies on hematological effects of meloxicam or other selective COX-2 inhibitors should be commenced. An extension of indications for administration of selective COX-2 inhibitors to those of myelosuppressions of various etiologies is desirable.
